# Intracortical and intercortical networks in patients after stroke: a concurrent TMS-EEG study

**DOI:** 10.1186/s12984-023-01223-7

**Published:** 2023-08-02

**Authors:** Zhongfei Bai, Jack Jiaqi Zhang, Kenneth N. K. Fong

**Affiliations:** 1 Department of Rehabilitation Sciences, The Hong Kong Polytechnic University, Kowloon, Hong Kong SAR; 2 Department of Rehabilitation, Shanghai YangZhi Rehabilitation Hospital (Shanghai Sunshine Rehabilitation Centre), School of Medicine, Tongji University, Shanghai, China

**Keywords:** Stroke, TMS-evoked potentials, Cortical excitability, Intracortical inhibition

## Abstract

**Background:**

Concurrent transcranial magnetic stimulation and electroencephalography (TMS-EEG) recording provides information on both intracortical reorganization and networking, and that information could yield new insights into post-stroke neuroplasticity. However, a comprehensive investigation using both concurrent TMS-EEG and motor-evoked potential-based outcomes has not been carried out in patients with chronic stroke. Therefore, this study sought to investigate the intracortical and network neurophysiological features of patients with chronic stroke, using concurrent TMS-EEG and motor-evoked potential-based outcomes.

**Methods:**

A battery of motor-evoked potential-based measures and concurrent TMS-EEG recording were performed in 23 patients with chronic stroke and 21 age-matched healthy controls.

**Results:**

The ipsilesional primary motor cortex (M1) of the patients with stroke showed significantly higher resting motor threshold (*P* = 0.002), reduced active motor-evoked potential amplitudes (*P* = 0.001) and a prolonged cortical silent period (*P* = 0.007), compared with their contralesional M1. The ipsilesional stimulation also produced a reduction in N100 amplitude of TMS-evoked potentials around the stimulated M1 (*P* = 0.007), which was significantly correlated with the ipsilesional resting motor threshold (*P* = 0.011) and motor-evoked potential amplitudes (*P* = 0.020). In addition, TMS-related oscillatory power was significantly reduced over the ipsilesional midline-prefrontal and parietal regions. Both intra/interhemispheric connectivity and network measures in the theta band were significantly reduced in the ipsilesional hemisphere compared with those in the contralesional hemisphere.

**Conclusions:**

The ipsilesional M1 demonstrated impaired GABA-B receptor-mediated intracortical inhibition characterized by reduced duration, but reduced magnitude. The N100 of TMS-evoked potentials appears to be a useful biomarker of post-stroke recovery.

**Supplementary Information:**

The online version contains supplementary material available at 10.1186/s12984-023-01223-7.

## Background

Transcranial magnetic stimulation (TMS) can transsynaptically excite pyramidal neurons by activating the excitatory and inhibitory interneurons located in layers II and III of the brain cortex [1]. A single pulse of TMS with adequate intensity applied to the primary motor cortex (M1) produces a neural transmission along the corticospinal descending pathway and evokes activity in the contralateral muscle –– activity that is termed motor-evoked potentials (MEPs) [1]. Therefore, TMS has been used as a tool for investigating the integrity of the corticospinal tract and the functional balance of excitatory and inhibitory circuits within the motor cortex.

Understanding post-stroke neurophysiological alteration using many TMS protocols have played an essential role in investigating the correlates of motor recovery with adaptive and maladaptive neurophysiological alteration, predicting prognosis, and guiding non-invasive brain stimulation for stroke. Compelling evidence indicates that corticospinal excitability of the contralesional M1 is comparable with that of healthy counterparts, but such is not the case for the excitability in the ipsilesional M1, which decreases with impaired contralateral motor function. The reduction of ipsilesional M1 excitability can be indexed by increased resting motor threshold (RMT) and decreased resting MEP amplitudes [2, 3]. By measuring short-interval intracortical inhibition (SICI) of the ipsilesional M1 of patients with stroke, paired-pulse studies have revealed dynamically changed GABA-A receptor-mediated intracortical inhibition. That inhibition is reduced at the acute stage post-stroke, which may imply a process of intracortical disinhibition, and it has returned to a normal level at the chronic stage [2]. GABA-B receptor-mediated intracortical inhibition has been extensively investigated, but studies have reported that long-interval intracortical inhibition (LICI, mediated by GABA-B receptors) either remained unchanged [4], reduced [5], or enhanced [6] in patients with stroke, leading to the question of how the GABAergic inhibitory circuits are altered after stroke?

Concurrent TMS and electroencephalography recording (TMS-EEG) is a novel approach that records the summation of postsynaptic excitatory and inhibitory potentials that occur in response to TMS pulses and are termed TMS-evoked potentials (TEPs). This approach enables assessments of local cortical excitability and connectivity between brain regions [7], and has been used to provide prognostic biomarkers that predict motor recovery after stroke [8]. Pharmacological studies have confirmed that the amplitudes of N45 and N100 are mediated by the GABA-A and GABA-B receptors, respectively [9, 10], thus establishing a close link between TEP peaks and GABAergic intracortical inhibition. In addition, TMS-EEG data offer a way to characterize impaired causal connectivity from the perturbed site to remote brain regions, in patients with neurological conditions [11]. Furthermore, by computing the phase synchronizations of neural oscillations, the cortical networks of possible interactions among different brain regions can be analyzed [12, 13].

A previous study in patients with acute stroke suggested that the absence of the N100 component was an indicator of severe motor impairment [8]. Another study showed that in a group of patients who were undergoing longitudinal recording, the TEP amplitudes decreased at the subacute stage post-stroke, and they gradually increased in parallel with motor recovery [14]. However, Gray et al. [15] found that the N100 amplitude in patients with chronic stroke was comparable with that in healthy controls, a finding that appears to differ from others obtained in the acute and subacute patient groups [8, 14]. Recently, researchers noted that different patterns of TEPs in terms of their morphologies could be observed separately from different neurological conditions and could even coexist within the same brain, depending on the site of stimulation [16, 17]. Specifically, stimulating the contralesional M1 of patients with stroke produced TEPs with small amplitudes, quickly changing deflections, and a complex spatial distribution, resembling those obtained from healthy awake individuals [16, 17]. TEPs with quickly changing deflections can be confirmed by inspecting whether ERSP in high-frequency bands (beta band) is retained [16]. In contrast, directly stimulating the perilesional region over the ipsilesional hemisphere has been found to produce TEPs that are characterized by large amplitudes, slow frequencies, and stereotypical EEG reactivity [16, 17] and are similar to those in healthy individuals during non-rapid eye movement sleep and in patients with unresponsive wakefulness syndrome [18]. Furthermore, slow-frequency TEPs seem to be associated with the severity of motor impairment due to stroke [17], and the occurrence of such TEPs is a pathological form of local cortical bistability [16]. However, it is noteworthy that the standard form of TEPs found in contralesional stimulation and healthy people can also be recorded in ipsilesional stimulation in patients with a small subcortical lesion [16, 17]. In view of possibly different mechanisms, the distinct morphologies of the TEPs that are identified in stroke should be disentangled carefully before any subgroup analyses and interpretations are conducted.

To the best of our knowledge, although previous studies with many TMS protocols have provided informative investigation into post-stroke neurophysiology, largely inconsistent findings were reported, particularly in GABA-B receptor-mediated intracortical inhibition. In addition to TEPs characterized by large amplitudes and slow frequencies, ipsilesional stimulation can also produce a TEP pattern similar to that in contralesional stimulation and healthy people, but the characteristics of this TEP pattern in terms of peaks, oscillations, and connectivity are inconclusive. The objective of this study, therefore, was to conduct a comprehensive neurophysiological examination of patients with stroke, using TMS-EEG and MEP-based measures to address previous inconclusive findings in patients with chronic stroke.

## Methods

### Participants

The study investigated twenty-three patients with a hemiparetic upper extremity due to first-ever unilateral cerebral stroke (age = 61.6 ± 6.9 years; 6 females; upper extremity section of the Fugl-Meyer Assessment = 60.3 ± 7.1). The inclusion criteria were patients who were (1) suffering from chronic stroke (time since stroke onset > 6 months); (2) right-handed; (3) and aged between 18 and 75 years. Patients with stroke were excluded if they met any of the following criteria: (1) had any contraindications to TMS [19]; (2) had known neurological disease excluding stroke; (3) had cognitive impairment (a score < 6/10 on the Abbreviated Mental Test) [20]; (4) or had uncontrolled hypertension (> 160/100 mm Hg). Detailed information about the patients with stroke is provided in Table 1. The initial lesion location was confirmed by radiological reports in their hospital records. Structural MRIs of 16 patients were acquired after they had enrolled in our study.

**Table 1:** Characteristics of the patients with stroke

Participant	Age (years)	Gender	Time since stroke (months)	Unaffected RMT	Affected RMT	Lesion type	Lesion location	FMA
Pt1	60	M	60	70	70	Ischemic	R lentiform region	60
Pt2	60	M	60	60	60	Hemorrhagic	R corona radiata and lentiform	63
Pt3	58	M	21	61	72	Hemorrhagic	L parietal and occipital region	66
Pt4	72	M	49	58	58	Hemorrhagic	L putamen	66
Pt5	59	M	62	60	80	Hemorrhagic	R frontoparietal lobe (M1, S1 involvement)	56
Pt6	63	M	72	56	58	Hemorrhagic	L lentiform	58
Pt7	67	M	49	54	62	Ischemic	L pons and parietal-occipital lobe	59
Pt8	53	F	82	50	60	Ischemic	R anterior temporal, insular, inferior frontal lobes, and putamen	65
Pt9	54	M	53	64	-	Hemorrhagic	L putamen	48
Pt10	63	F	117	68	81	Hemorrhagic	L thalamus and posterior limb of the internal capsule	59
Pt11	63	M	53	65	78	Ischemic	L corona radiata and putamen	63
Pt12	72	M	176	66	68	Ischemic	R posterior limb of the internal capsule	61
Pt13	69	M	312	50	55	Ischemic	R anterior limb of the internal capsule	66
Pt14	54	M	10	70	80	Ischemic	L corona radiata	60
Pt15	62	F	69	64	76	Ischemic	L basal ganglia	60
Pt16	61	F	57	68	76	Hemorrhagic	L corona radiata, posterior limb of the internal capsule, thalamic regions	60
Pt17	63	M	120	55	55	Hemorrhagic	R lentiform and external capsule	66
Pt18	68	M	44	64	56	Ischemic	R basal ganglia	63
Pt19	67	M	11	80	80	Ischemic	L inferior frontal gyrus	66
Pt20	61	F	47	62	62	Ischemic	L midbrain	66
Pt21	51	M	96	66	68	Ischemic	R frontal lobe and pons	66
Pt22	71	M	166	65	78	Ischemic	R posterior limb of the internal capsule	35
Pt23	46	F	54	82	78	Ischemic	L temporal, frontal and parietal lobes	55

F = female; M = male; RMT = resting motor threshold; L = left; R = right; M1 = primary motor cortex; S1 = primary somatosensory cortex; FMA = Fugl-Meyer Assessment for upper extremity

Twenty-one age-matched healthy participants without any known history of neurological or psychiatric diseases (and who were right-handed; age = 60.9 ± 6.9 years; seven females) were recruited as healthy controls. This study was conducted following the ethical principles of the Declaration of Helsinki [21], and was approved by the Human Research Ethics Committee of the Hong Kong Polytechnic University (Reference Number: HSEARS20200621001). Written informed consent was obtained from all participants.

### Study design

In this cross-sectional study, a battery of MEP-based measures and TMS-EEG were administered to both hemispheres of each participant. White noise masking was applied to minimize contamination from auditory-evoked potentials elicited by TMS click sounds [22]. Two control conditions were completed to evaluate whether our TMS-EEG setups were appropriate for minimizing the contamination of auditory-evoked potentials from TEPs (see details in the Additional File).

### TMS

Participants were seated in a TMS-specific adjustable chair with head and back supports and were kept awake with their eyes open. To maintain consistency throughout the experiment, all TMS procedures were performed over an EEG cap. Biphasic TMS pulses were always delivered to the hotspot of the first dorsal interosseous muscle, using a figure-of-eight cooling coil (Cooling B-65, external diameter of each wing: 75 mm) connected to a magnetic stimulator (model X100, MagVenture A/S, Denmark). The motor hotspot was defined as the position at which the largest and most reliable MEPs could be obtained from the first dorsal interosseous muscle contralateral to stimulation. The MEPs were recorded from corresponding muscles using disposable Ag-AgCl surface electrodes positioned in a belly-tendon montage, and a ground electrode was placed on the ulnar styloid process. The MRI-less version of a TMS-navigation system (Localite, Bonn, Germany) was used to guide the TMS coil positioning, in which a standard magnetic resonance imaging (ICBM152) was warped to match the individual’s brain. To effectively stimulate the M1, the coil was placed on the scalp at approximately 45° away from the midline, with the handle pointed backwards and laterally.

RMT was defined as the minimum intensity (measured as the % of the maximal stimulator output) that could elicit peak-to-peak MEP amplitudes higher than 50 µV in at least five out of 10 trials. Four MEP-based measures were recorded and analyzed: the cortical silent period (cSP), MEPs, intracortical facilitation (ICF), and SICI. Eight trials were recorded for each protocol, with inter-trial intervals ranging from 4 to 5 s. The intensity of test pulses was fixed at 120% of the RMT for all MEP-based measures. The cSP was the disruption of background electromyogram (EMG) activity by a suprathreshold test pulse while sustaining 30% of the maximal voluntary strength of thumb-index finger contraction. The amplitude of single-pulse MEPs was used to measure corticospinal excitability at rest. The SICI and ICF were obtained by delivering a suprathreshold test pulse after a subthreshold conditioning pulse at 80% of the RMT, with inter-pulse intervals of 2 ms and 10 ms, respectively. When measuring RMT, single-pulse MEP, SICI, and ICF, the participants were instructed to relax their hand muscles, with a background EMG amplitude around 10 µV.

The raw signals of MEP-based measures were recorded by a bipolar channel of an EEG system (SynAmps, NeuroScan, USA), digitized at 5 kHz, and stored on a laptop for offline analysis.

### TMS-EEG recording

With referenced to previous studies [16, 17], TMS-EEG recording was carried out using a TMS-compatible DC EEG system (SynAmps, NeuroScan, USA) with 64 Ag/AgCl electrodes mounted according to the international 10–10 system. The raw signals were online-referenced to FCz, grounded to AFz, digitized at a sampling rate of 5 kHz, and online-filtered below 2 kHz. The impedance between the scalp and the electrodes was maintained below 5 kΩ to optimize the signal-to-noise ratio. During recording, 90 TMS pulses were applied to the M1, with intertrial intervals of 4 to 5 s. The intensity for the healthy participants was set at 110% of the RMT of the corresponding M1. The stimulation intensity for both hemispheres of the patients was 110% of the RMT of the contralesional M1. Because the ipsilesional MEPs of Patient 9 (Pt9) were not sufficiently large, we located the ipsilesional M1 using his T1-weighted MRI (resolution: 0.8*0.8*0.8mm^3^). To suppress auditory-evoked potentials produced while the coil discharged, all participants wore an inserted earphone, and white noise was played [7]. The volume of the noise was as loud as it could be for all participants. To minimize TMS-decay artifacts, a thin piece of foam (3 mm thick) was placed underneath the coil to prevent direct contact with the electrodes [7], and the direction of lead wires near the coil was rearranged so that they were perpendicular to the coil [23]. To reduce eye movements, participants were required to gaze at a black cross with a white background, approximately 2 m away.

### Data analysis

The signals of EMG and EEG were preprocessed offline using EEGLAB 14.1.2 [24], TESA extension [25], FieldTrip [26]. The analyses were performed with reference to others [7, 16, 27–29]. Details of EMG data analysis and EEG preprocessing can be found in the Additional File.

In the temporal domain, we defined five peaks, with reference to the previous literature [7]: P30 (28–35 ms), N45 (40–50 ms), P65 (55–75 ms), N100 (90–130 ms), and P180 (160–220 ms). The above definition was not applicable for the two patients whose TEPs were characterized by large initial amplitudes and slow frequencies and did not present unambiguous quick peaks. Global mean field power (GMFP) of the TEPs was computed using the following formula to explore the global reactivity following TMS pulses [27]:

1 $$\text{G}\text{M}\text{F}\text{P}\left(t\right)= \sqrt{\frac{\left[\sum _{i}^{k}{({V}_{i}\left(t\right)-{V}_{mean}(t\left)\right)}^{2}\right]}{K}}$$

Where *t* is time, *V* is the voltage at channel *i*, and *K* is the number of channels. To quantify the complexity of brain responses to TMS pulses, the perturbational complexity index-state transitions (PCI-st) was calculated by using dimensionality reduction and state transitions quantification [30].

In the time-frequency domain, event-related spectral perturbation (ERSP) was computed by decomposing individual trials using the Morlet wavelet transform (three cycles, a frequency step of 1 Hz between 4 and 48 Hz, baseline-corrected [− 625 – −100 ms], time resolution of ~ 3 ms) and then averaging across trials. In accordance with a recent study in which patients with slow and local deflections presented with significantly attenuated ERSP at the late stage following TMS pulses [16], we also defined early (15–150 ms) and late (150–350 ms) stages for subsequent analysis. The ERSP values were averaged in four predefined frequency bands of interest: theta (4–8 Hz), alpha (8–13 Hz), beta-1 (13–20 Hz), and beta-2 (20–30 Hz).

In the current study, ipsilesional stimulation induced significantly suppressed ERSP over the midline-frontal and parietal regions of the stimulated hemisphere at the early stage. Therefore, we further investigated functional connectivity and whole brain networks at that early stage. The connectivity measure of interest used in our study was the debiased weighted phase lag index (dwPLI), which was weighted by the magnitude of the imaginary component of cross-spectrum and insensitive to noise and volume conduction [28]. To obtain the dwPLI, first, the EEG signals after preprocessing were filtered to the frequency band of interest by a finite impulse response filter. Second, the Hilbert transform was employed to obtain the instantaneous phase of the signals. Third, the dwPLI was calculated and averaged at the early stage (15–150 ms). Four regions of interest were predefined: the stimulated primary motor cortex (M1), the nonstimulated M1, the frontal region, and the parietal region. Taking right stimulation, for example, the dwPLIs of three channel pairs (C4-F2, C4-F4, C4-AF4) were averaged to denote the functional connectivity between the M1 and frontal regions, and two channel pairs (C4-P2, C4-P4) were averaged for the functional connectivity between the M1 and parietal regions. The C4-C3 channel pair served as the functional connectivity between the bilateral M1. For the whole-brain connectivity, two network measures, weighted transitivity and weighted global efficiency, were calculated using the graph theory and employing the Brain Connectivity Toolbox [29]. Respectively, weighted transitivity and weighted global efficiency are the ratio of triangles to triplets and the average of the inverse shortest path length in a network, weighted by edge coefficients, and reflecting functional segregation and integration of the brain network. To construct a network without spurious connectivity weights, the dwPLI matrix was thresholded by preserving 5–20% of the strongest connectivity with steps of 0.5%, resulting in the same amount of connectivity across participants at a given threshold. Subsequently, the two network measures were computed at each threshold. Finally, the areas under the curves (AUC) of weighted transitivity and weighted global efficiency were obtained by computing their integrals across these multiple thresholds using the trapezoidal method.

### Statistical analyses

Statistical analyses were performed using SPSS22 (IBM, NY, USA) and FieldTrip in MATLAB 2016a. The alpha threshold was set at 0.05 (two-tailed). There was no significant difference on any outcomes between both hemispheres in the healthy controls (see details in the Additional File). Therefore, the left and right hemispheric measures from the healthy controls were averaged to obtain merged values for further between-group comparisons.

The first set of analyses was within-group comparisons using paired *t*-tests. The second set of analyses was between-group comparisons made by independent *t*-tests. To reduce type I error, Bonferroni correction was applied (corrected alpha threshold = 0.05/3, with three as the number of comparisons).

Non-parametric cluster-based permutation tests (number of permutations: 10,000) with the Monte Carlo method were conducted to address the multiple-comparisons problem in spatial and temporal dimensions when examining the within- and between-group differences in TEPs and TMS-related oscillations [31]. Any positive or negative clusters with *P*-values < 0.025 were considered to be significantly different. To further elaborate the mechanism of N100 in patients with stroke, first, the local mean field power (LMFP) from 90 to 130 ms surrounding the ipsilesional M1 was averaged across the time window. Second, we explored the Pearson’s correlation coefficients between the LMFPs of the N100, MEP-based measures, and AUCs of transitivity and efficiency.

## Results

Twenty-one of the patients with stroke presented ipsilesional TEPs with quickly changing deflections and complex spatial distribution, whereas the ipsilesional TEPs of the remaining two patients (Pt22 and Pt23) were characterized by large amplitudes, slow frequencies, and stereotypical EEG reactivity. Therefore, those two patients were not included in our subsequent subgroup analysis of EEG measures (*n* = 21). Because the ipsilesional MEPs of Pt9 were not recordable, he was not included in the analysis of MEP-based measures (*n* = 20).

### MEP-based measures

**Fig. 1:**
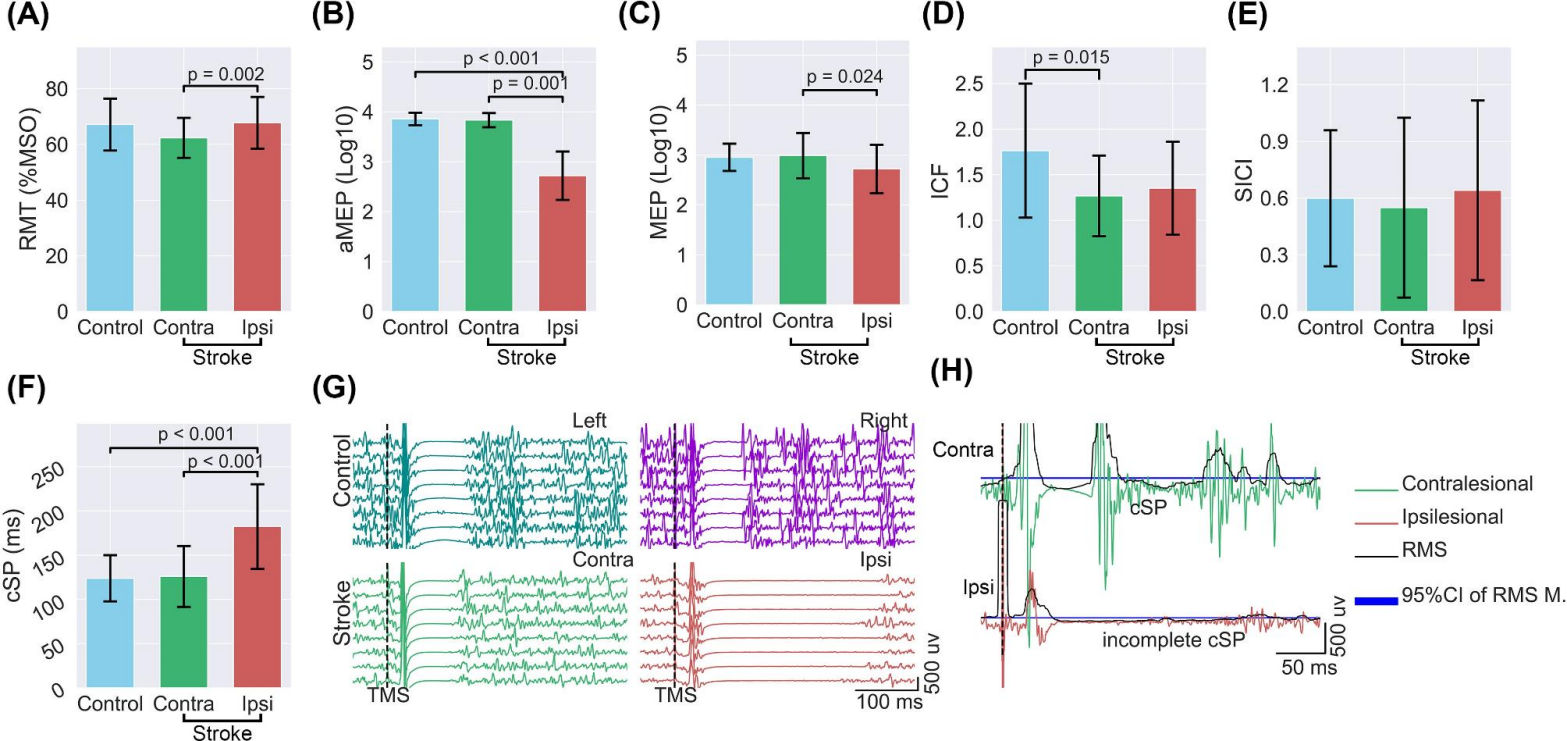
MEP-based measures in patients with stroke and healthy controls. Overall, ipsilesional corticospinal excitability is reduced, shown by enhanced RMT and reduced MEP amplitudes. The duration of ipsilesional cSP is prolonged. Pt9, whose ipsilesional MEPs were not recordable, was excluded from the analysis of MEP-based measures. (**A** – **F**) Comparisons of the ipsilesional and contralesional RMTs, aMEPs, MEPs, ICF, SICI, and cSP of the patients with stroke and the measures of the healthy controls. (**G**) Unambiguous silent periods of a healthy participant and a patient with stroke, showing eight trials of each measure. (**H**) An incomplete ipsilesional cSP was found in eight patients with stroke, exemplified by slight and persistent EMG activity after aMEPs until EMG bursts returned. Notes: RMT: resting motor thresholds; MSO: maximal stimulation output; MEP: motor-evoked potential; aMEP: active MEP; ICF: intracortical facilitation; SICI: short-interval intracortical inhibition; cSP: cortical silent period; RMS: root mean square of baseline EMG activity

In the patients with stroke, the ipsilesional M1 had a significantly larger RMT (*t* = 3.61, *P* = 0.002) than their contralesional M1 did, but independent *t*-tests indicated that neither ipsilesional (*t* = 0.21, *P* = 0.834) nor contralesional (*t* = − 1.78, *P* = 0.083) RMT was significantly different from that of healthy controls (Fig. 1). The active MEP amplitude in the ipsilesional M1 of the patients with stroke was significantly smaller than that in the contralesional M1 (*t* = − 4.08, *P* = 0.001) and in the M1 of the healthy controls (*t* = − 4.48, *P* < 0.001), but the active MEP amplitude in the contralesional M1 of the patients with stroke did not significantly differ from that in the M1 of the healthy controls (*t* = − 0.64, *P* = 0.523). Likewise, there was a slight reduction of the ipsilesional MEP amplitude compared with that in the contralesional M1 (*t* = − 2.45, *P* = 0.024), but the difference was not significant after Bonferroni correction. Neither the ipsilesional (*t* = − 1.87, *P* = 0.068) nor the contralesional (*t* = 0.29, *P* = 0.771) MEP amplitude was significantly different from that of healthy controls. The contralesional ICF was significantly lower than that of healthy controls (*t* = − 2.57, *P* = 0.015), but that of the ipsilesional M1 was not significant after Bonferroni correction (*t* = − 2.04, *P* = 0.049); in addition, the ICF comparison between the bilateral M1 was not significant (*t* = 0.47, *P* = 0.644) in the patients with stroke. The comparison of the SICI values did not yield any significant differences (all *P* > 0.05). An unambiguous cSP was identified in all of the healthy controls and 12 out of 20 patients with stroke in the ipsilesional M1. However, the remaining eight patients presented low-level of EMG activity during the silent period (Fig. 1G and H). The contralesional cSP was not significantly different from that of the healthy controls (*t* = 0.22, *P* = 0.826). Nevertheless, the ipsilesional cSP was significantly prolonged compared with the contralesional cSP (*t* = 4.55, *P* < 0.001) and the cSP of the healthy controls (*t* = 4.70, *P* < 0.001). A correlation analysis between the bilateral difference in RMT and the bilateral difference in cSP was carried out, and a nonsignificant correlation was found (*r* = 0.26, *P* = 0.260), thus ruling out the possibility that the prolonged cSP was related to an increase in the intensity of stimulation.

### TMS-evoked potentials

**Fig. 2:**
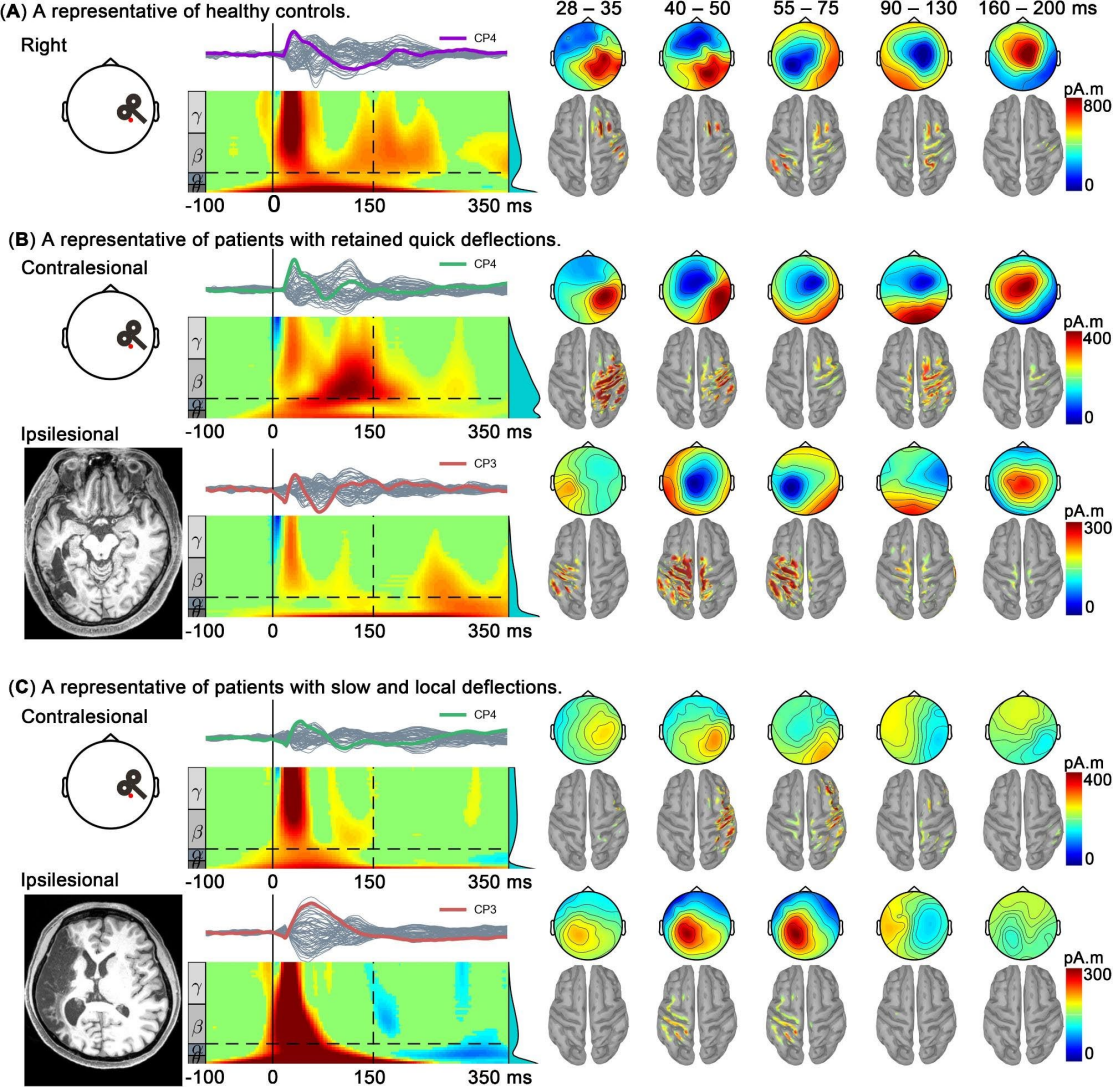
Data from a representative healthy participant and from two representative patients with stroke who had distinct, different TEP deflection patterns. (**a**) Right TEPs of the healthy participant. (**B** – **C**) The patients’ contralesional TEPs are shown in the top row and their ipsilesional TEPs are in the bottom row of each panel, for (**B**) the representative patient with retained quick deflections, and (**C**) the representative patient with slow and local deflections. The stroke lesions of the two patients are shown in their T1-weighted images. Butterfly plots of TEPs and ERSP of a channel (CP4 or CP3, highlighted in time series, located at the red dots) close to the TMS stimulation are shown in the left. In the ERSP plot, a bootstrap method (alpha threshold: 0.05) was used to identify significantly increased (event-related synchronization, colored in yellow/red) or decreased (event-related desynchronization, colored in blue) ERSP compared with the baseline (− 625 ms to − 100 ms); nonsignificant time-frequency bins are colored in green. On the right side of the figure, the topographies and estimated sources (thresholded at 50%) are shown for five windows corresponding to five well-known peaks of TEPs. The source estimation was carried out by using the minimum norm estimation method using the Brainstorm toolbox

In the healthy controls, single TMS pulses elicited a series of quickly changing deflections, which originated from the stimulation site and then propagated to surrounding and distant brain areas, as shown by the source estimation (Fig. 2). In the patients with stroke (TEPs of each patient are shown in Additional File), contralesional TMS stimulation also elicited sustained responses that were similar to those obtained from the healthy controls and were in line with the findings of previous studies [16, 17]. However, two distinct patterns of TEPs obtained from ipsilesional M1 stimulation were identified. We found that 21 of the patients with stroke retained quick deflections that were similar to those observed in the healthy controls. In contrast, the TEPs of the two remaining patients were characterized by slow frequencies and large local amplitudes, lasting from 30 ms to roughly 100 ms, and they were not included in subsequent statistical analysis. Source estimation indicated that their activation at the early stage (15–150 ms) was always localized at the stimulation site and was eliminated almost completely during the late stage (150–350 ms). Such TEPs with slow early peaks have been well reported [16, 17]. Our present study therefore focused on the patients with quickly changing deflections and investigated how they differed from healthy controls in the temporal and time-frequency domains of TEPs.

**Fig. 3:**
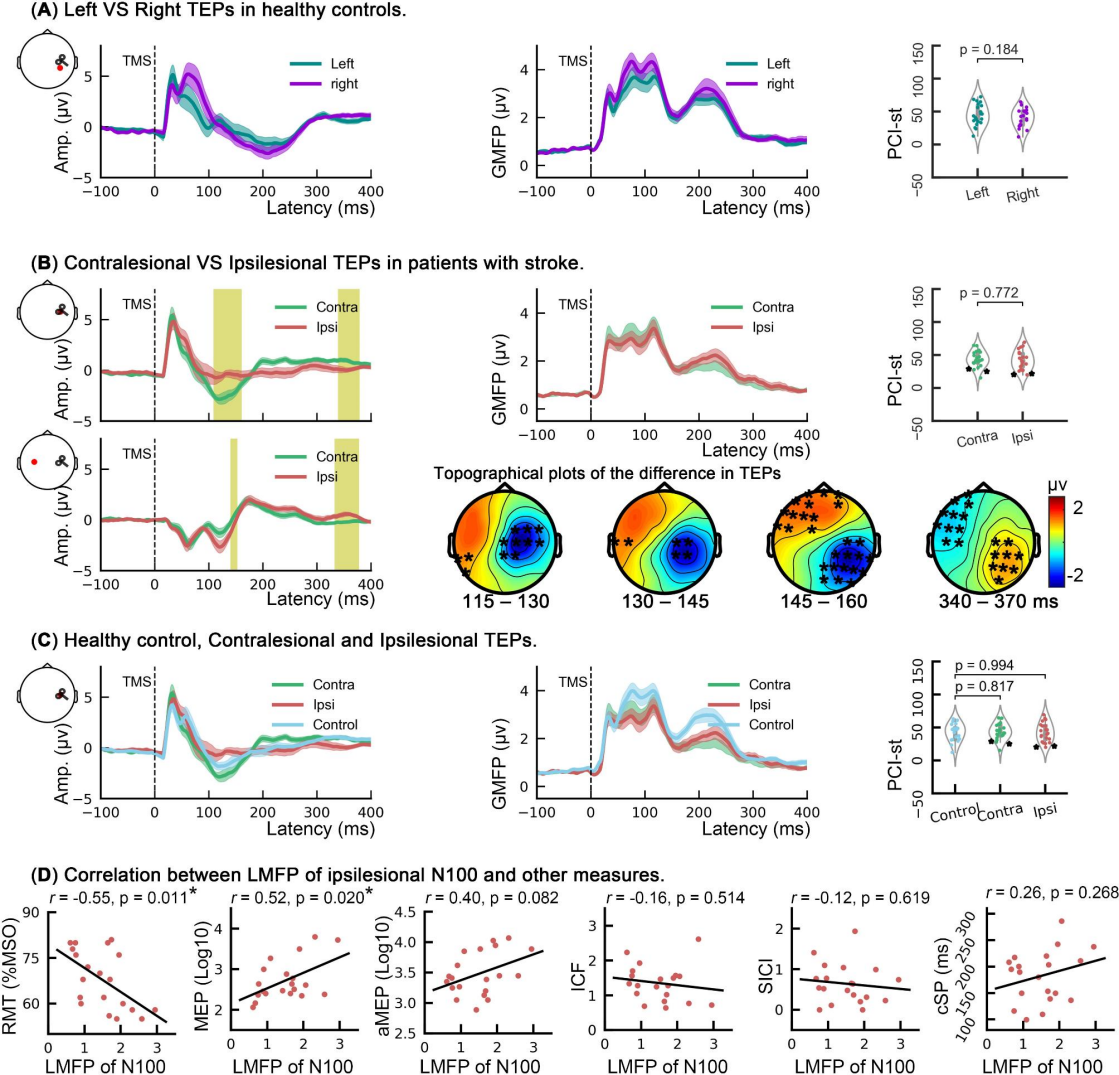
Analyses of the TEPs. (**A**) Comparison of the left and right TEPs in healthy controls. The first and second plots show the TEP amplitudes of a representative channel (CP4, located at the red dot) and the GMFP of the TEPs. The third plot is the comparison of left and right PCI-st. (**B**) Comparison of the contralesional and ipsilesional TEPs in patients with stroke. The first plot of each row is the representative channel located at the right (C4) and left (C3) hemispheres. Yellow rectangles indicate time windows in which significant differences between the ipsilesional and contralesional hemispheres were found. In addition, these differences are shown in four topographies. In the topographies, the black asterisk (*) represents significant clusters found in cluster-based permutation tests. The GMFP and PCI-st plots are also shown. The * in the PCI-st plot represents the values of two patients with slow and local TEPs. (**C**) Comparison of the TEPs in the patients with stroke and healthy controls. In short, no significant difference was found in any measure. (**D**) Correlations between the LMFP of the ipsilesional N100 and other measures. Notes: The curve shadings are the means ± standard errors. TEPs: TMS-evoked potentials; GMFP: global mean field power; PCI-st: perturbational complexity index-state transitions; LMFP: local mean field power; RMT: resting motor threshold; MSO: maximal stimulation output; MEP: motor-evoked potential; aMEP: active motor-evoked potential; ICF: intracortical facilitation; SICI: short-interval intracortical inhibition; cSP: cortical silent period

As shown in Fig. 3, ipsilesional stimulation produced TEPs with significantly smaller N100 amplitudes than those from contralesional stimulation around the stimulated hemisphere (*P* = 0.007), whereas larger amplitudes were found over the central and prefrontal regions of the nonstimulated hemisphere (*P* = 0.012), thus suggesting that the N100 amplitude over the ipsilesional hemisphere was always lower than that of the contralesional hemisphere, irrespective of the hemispherical sides of stimulation. In addition, reversed differences between the above two stimulation conditions were found at roughly 340 ms to 370 ms post-TMS pulses. Regarding the comparison between populations, cluster-based permutation tests (independent *t*-tests) failed to show any significant difference in TEP amplitudes. The PCI-st of the brain responses to TMS pulses applied to both hemispheres of the patients with stroke were comparable (*t* = 0.29, *P* = 0.772), and they did not significantly differ from healthy controls (all *P* > 0.05). The LMFP of the ipsilesional N100 was significantly associated with RMT (*r* = − 0.55, *P* = 0.011) and MEPs (*r* = 0.52, *P* = 0.020), but it was not significantly associated with active MEPs, ICF, SICI or cSP (all *P* > 0.05).

### TMS-related oscillatory power

**Fig. 4:**
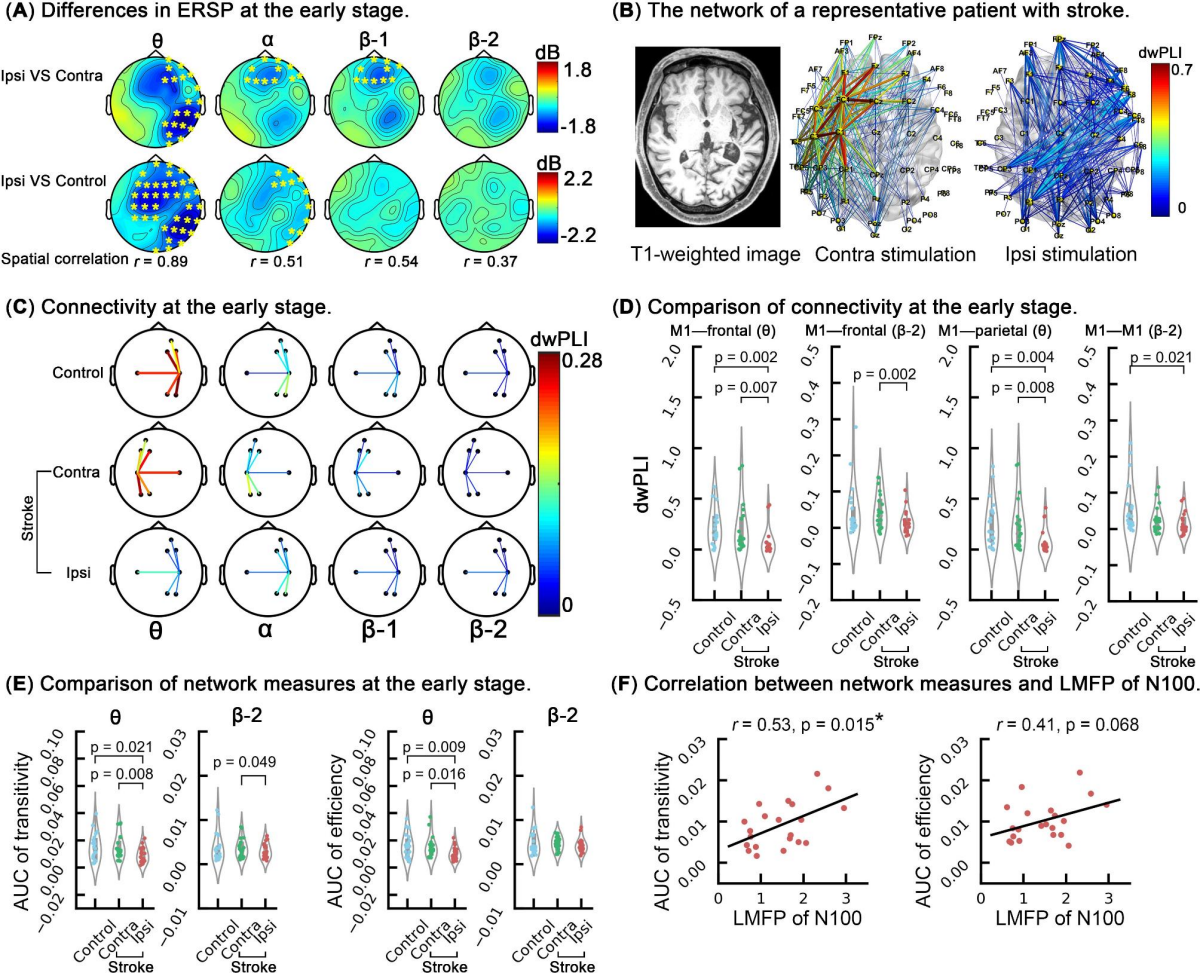
Analyses of the ERSP. (**A**) Differences in ERSP at the early stage (15–150 ms). Topographies in the first row show the differences between the patients’ ipsilesional and contralesional ERSP, and topographies in the second row show the differences in the ERSP derived from ipsilesional stimulation in the patients and M1 stimulation in the healthy controls. The asterisks (*) represent significant clusters found in cluster-based permutation tests. Spatial correlation was obtained by performing a Pearson product-moment correlation analysis for the above two sets of differences in the theta (*r* = 0. 89, *P* < 0.001), alpha (*r* = 0.51, *P* < 0.001), beta-1 (*r* = 0.54, *P* < 0.001) and beta-2 (*r* = 0.37, *P* = 0.004). (**B**) The network in a representative stroke patient. (**C**) Connectivities at the early stage. The values are the average values of 21 patients. (**D**) Comparison of the connectivities at the early stage. (**E**) Comparison of network measures at the early stage. (**F**) Correlations between the measures of network transitivity and the LMFP of N100 and measures of network efficiency and the LMFP of N100. Theta was the only frequency band in which significant differences in connectivity and network measures were found. Therefore, the correlation between network measures in the theta band and the LMFP of N100 was explored. Note: ERSP: event-related spectral perturbation; dwPLI: debiased weighted phase lag index; AUC: area under curve; LMFP: local mean field power

Cluster-based permutation tests revealed that at the early stage (15–150 ms), ipsilesional stimulation induced significantly suppressed broadband ERSP in the theta (*P* < 0.001), alpha (*P* = 0.003), and beta-1 (*P* = 0.005) frequency bands compared with ERSP produced by contralesional stimulation (Fig. 4). The significant differences were primarily over the midline-prefrontal and parietal regions of the stimulated hemisphere, but the ERSP located at the ipsilesional M1 was not significantly different from that at the contralesional M1. Similarly, compared with healthy controls, ipsilesional stimulation in the patients with stroke also showed significantly suppressed ERSP in theta (*P* = 0.001) and alpha (*P* = 0.013) frequency bands at the early stage over the stimulated hemisphere. The comparisons of TMS-related oscillations at the late stage (150–350 ms) did not yield any significant differences (data not shown).

At the early stage following TMS pulses, the connectivity in the theta band between the patients’ ipsilesional M1 and prefrontal region was significantly lower than that of the healthy controls (*t* = − 3.30, *P* = 0.002) and that of the contralesional hemisphere (*t* = − 2.98, *P* = 0.007). Similarly, significantly lower connectivity was also found in the beta-2 band (*t* = − 3.47, *P* = 0.002). For the connectivity between the ipsilesional M1 and the parietal region in the theta band, it was significantly lower that that of the contralesional hemisphere (*t* = − 2.93, *P* = 0.008) and that of the healthy controls (*t* = − 3.09, *P* = 0.004). Ipsilesional M1 stimulation produced lower M1–M1 connectivity than that of the healthy controls (*t* = − 2.46, *P* = 0.021), but it was not significant after Bonferroni correction.

Furthermore, network analysis showed that the AUC of weighted transitivity in the theta band produced by ipsilesional stimulation was significantly lower than that produced by contralesional stimulation (*t* = − 2.97, *P* = 0.008), and tended to be lower than that of the healthy controls (*t* = − 2.43, *P* = 0.021). Similarly, the AUC of weighted transitivity in the beta-2 band produced by ipsilesional stimulation tended to be lower than that produced by contralesional stimulation (*t* = − 2.09, *P* = 0.049). In addition, ipsilesional stimulation produced a significantly lower AUC of weighted efficiency in the theta band in the patients with stroke than contralesional stimulation did (*t* = − 2.63, *P* = 0.016) and than that of the healthy controls (*t* = − 2.76, *P* = 0.009). However, no significant difference in the AUC of weighted efficiency in the beta-2 band (*t* = − 1.31, *P* = 0.206) was found. Pearson’s correlation analysis showed that the LMFP of N100 was significantly correlated with the AUC of weighted transitivity (*r* = 0.53, *P* = 0.015), but it was not significantly correlated with the AUC of weighted efficiency in the theta band (*r* = 0.41, *P* = 0.068).

## Discussion

In line with previous findings [16, 17], two different TEP patterns were replicated in patients with chronic stroke with mild motor impairment, based on their morphologies. We then demonstrated reduced corticospinal excitability and prolonged cSP in the ipsilesional hemisphere of stroke patients with quickly changing TEPs. The amplitude of the N100 component around the ipsilesional M1 was lower than that in the contralesional M1, irrespective of the hemispheric sides of stimulation, and it was also found to be correlated with the MEP-based measures of corticospinal excitability. Therefore, these findings indicate an impairment of intracortical excitatory and inhibitory functioning in patients with chronic stroke. Moreover, ipsilesional stimulation induced significantly suppressed broadband ERSP over the midline-prefrontal and parietal regions, accompanied by reduced connectivity and brain network properties, thus suggesting an impairment of the connectivity between the motor and frontoparietal networks.

As shown in Fig. 2, the first type of TEPs produced by ipsilesional M1 stimulation is characterized by small amplitudes in the temporal domain and retained event-related synchronization in the time-frequency domain. On the contrary, ipsilesional TEPs of two patients present large initial and reduced late amplitudes in the temporal domain and event-related desynchronization in high frequency bands. The latter type can be physiological in healthy people during non-rapid eye movement sleep and pathological in patients with unresponsive wakefulness syndrome [18]. Regarding its underlying mechanism, the occurrence of this latter types of TEPs probably indicates a silent and hyperpolarized state in cortical neurons (also known as off-period), caused by local excitation/inhibition imbalance and cortico-cortical disfacilitation due to white matter disruption [16].

### Impairment of GABA-B receptor-mediated inhibition

Overall, our findings on MEPs, ICF, and SICI were consistent with a recent meta-analysis [2]. Nevertheless, non-significant difference on RMT was found between the ipsilesional M1 and healthy controls, probably due to relatively low-level of motor impairment (FMA = 60.3 ± 7.1). We found two patterns of cSP, differentiated by whether or not muscle activity during the silent period was suppressed completely. For the incomplete cSP, low-level EMG activity can be observed in the silent period, which may be an indicator of excessive spinal reflex in patients with stroke [32, 33]. Since the early and late parts of cSP are due to spinal and intracortical mechanisms [34], respectively, another explanation for the persistent low-level EMG during cSP is that it may be a sign of a reduced magnitude of intracortical inhibition mediated by GABA-B receptors. By using different methods to define cSP, our study showed an overall prolonged cSP in the ipsilesional M1 in patients with stroke. A prolonged cSP found in the ipsilesional M1 has been thought to be caused by increased activity of GABA-B receptor-mediated inhibitory circuits in the cortex [35]. However, another GABA-B receptor-mediated inhibitory biomarker, LICI, has been found to be reduced in stroke, which seems incompatible with the studies using cSP [5].

The N100 is a robust biomarker of intracortical inhibition mediated by GABA-B receptors, and its underlying mechanism is similar to that of LICI, as suggested by previous pharmacological-TMS experiments [9, 10]. In the current study, we found that the N100 amplitude derived from ipsilesional stimulation was significantly lower than that from contralesional stimulation, and it was focally distributed around the stimulated site. Therefore, this finding implied reduced intracortical inhibition in the ipsilesional M1, and the prolonged cSP may reflect a different aspect of GABA-B receptor-mediated inhibition, with two possible explanations. First, our correlation analysis showed that the N100 was not associated with cSP. Second, an oral dose of baclofen can result in increased LICI but has no effect on cSP [36]. Tiagabine can prolong cSP [37], but has no effect on the amplitude of TEPs [38], thus suggesting that cSP is not a biomarker for the magnitude of GABA-B receptor-mediated inhibition, and it may only reflect the duration of GABA-B receptor-mediated inhibition. It is noteworthy that N100 in TEPs and LICI are obtained at a resting state, while cSP represents the intracortical inhibition and spinal inhibition during isometric contraction, probably accounting for some degree of dissociation between cSP and N100 found in the current study [36]. For the underlying mechanism of the alteration of GABA-B receptor-mediated inhibition in stroke, animal studies have suggested that the expression of GABA-B receptors can decrease in the cortex and subcortical structures after stroke [39]. On the other hand, the amount and efficiency of GABA transporters could continuously decrease in the peri-lesion cortex [40, 41]. As a result, phasic GABA-B release from the presynaptic terminal may activate only a limited number of GABA-B receptors, and that in turn results in a reduced magnitude of GABA-B receptor-mediated inhibition. The released GABA cannot be removed promptly due to the downregulation of GABA transporters, which may explain the prolonged cSP.

In contrast, recent studies using a threshold hunting method have reported excessive LICI in both the ipsilesional and contralesional M1 of patients with stroke [6, 42]. For that method, the theory that high intracortical inhibition requires high-intensity test pulses to evoke comparable MEP amplitudes is quite reasonable. However, we have a concern regarding its implementation –– specifically, the intensity of test pulses can be 20–30% higher than that of nonconditioned test pulses [6], and this intensity finally can be 130–140% of the ipsilesional RMT. Di Lazzaro et al. [43] have shown that TMS at a high intensity excites not only superficial interneurons but also corticospinal fibers in the white matter [43]. Therefore, the test pulse of the threshold hunting method may excite the pyramidal neuron directly rather than transsynaptically, likely leading to an incorrect readout of intracortical inhibition. This notion has also been supported by another study in which the conditioning pulse had weaker inhibitory, or even facilitatory, effects on test pulses with increased intensity [44].

### Impaired connectivity between the motor and frontoparietal networks

The neocortex is interconnected by white matter, forming direct corticocortical connections and indirect connections (nonreciprocal cortico-thalamocortical circuits). Brain oscillations reflect the transmission and processing of endogenous information within a network through synchronization in specific frequency bands. For instance, long-range synchronization in the theta band mediates the connectivity between the prefrontal cortex and the posterior parietal cortex within the frontoparietal network [45]. Rosanova et al. [46] documented that TMS-related oscillations can be preserved while remote and connected brain areas are being stimulated, thus implying the significance of TMS-EEG in examining the integrity of long-range connectivity. In the present study, our analysis failed to identify significantly different TEP amplitudes in the prefrontal and parietal regions. Interestingly, a suppressed TMS-related oscillatory spectrum in a broadband range was noted in these regions, in parallel with a disruption of connectivity. It has been shown that TMS-related signal propagation in the brain is highly associated with structural networks rather than with functional networks [47]. Therefore, our findings may reveal impaired long-range intra-/interhemispheric corticocortical connectivity between the motor network and distant networks (i.e., the frontoparietal network) in patients with chronic stroke [48–50]. Our speculation is supported by previous diffusion tensor imaging studies in which Wallerian degeneration presented not only in the ipsilesional corticospinal tract, but also in the superior longitudinal fasciculus, corpus callosum, and inferior fronto-occipital fasciculus [51–53]. Notably, degeneration of the superior longitudinal fasciculus within the frontoparietal network has been found to be associated with impaired executive function in patients with chronic stroke [54]. Overall, the lesions of most patients in the present study were in subcortical structures rather than in the prefrontal or parietal regions. Therefore, the suppressed TMS-related oscillatory spectrum over the prefrontal and parietal regions may indicate secondarily impaired structural networks, beyond the motor network. However, it is also possible that the endogenous properties of the prefrontal/parietal corticothalamic circuits are changed at the chronic stage following stroke. Therefore, multi-site TMS-EEG recordings will be necessary to test the notion of secondary structural-network impairment.

From the perspective of network topology, a brain network with small-world characteristics supports the efficient transformation of information. A previous study showed bilaterally decreased small-world characteristics in the delta band in patients at the acute stage of stroke [55], and the characteristics in certain frequency bands may be predictive for motor recovery [56]. As a result of training, small-worldness can be increased, in parallel with motor improvement [57]. By using graph theory-based network measures in the present study, we found that ipsilesional stimulation produced significantly lower segregation and integration than contralesional stimulation did, and than that of the healthy controls. Therefore, our findings suggest impaired small-world brain networks in patients with chronic stroke. Additionally, we found a positive correlation between these network measures and the LMFP of the N100. When considered in conjunction with another finding that the LMFP of the N100 was also positively associated corticospinal excitability, our study supports the notion that the N100 may be a meaningful biomarker of brain function following stroke [8].

Several limitations should be acknowledged. First, there is no consensus on how to analyze the duration of cSP, therefore, the approach of extracting the time from TMS pulse onset to returned EMG bursts, was used in the current study, which might limit the comparability with previous studies. Second, suprathreshold intensity was used for TMS-EEG, which may produce somatosensory-evoked potentials due to the re-afferent feedback from peripheral muscle twitches [58]. Although late components of somatosensory-evoked potentials, such as P100, are mainly over the bilateral secondary somatosensory cortex, our current study can not completely estimate the extent of their contamination to our findings on N100. To avoid the above issue, future studies may use subthreshold intensity for TMS-EEG recording. Third, a realistic sham condition was not employed. Our control conditions showed that noise masking was effective in reducing the N100 amplitude of auditory-evoked potentials, but more or less residual potentials can be found in some participants. Our within-participant comparisons can eliminate the influence of auditory-evoked potentials to TEPs, this is not applicable for between-participant comparisons, which may have weakened the validity of between-participant comparison. Therefore, further studies can employ a realistic sham condition that mimics the somatosensory and auditory stimuli while TMS discharges. Then, evoked potentials in the realistic sham condition were subtracted from mixed TEPs to obtain clean TEPs [59]. Fourth, the current study included a non-homogeneous cohort including patient with a stroke lesion at the cortex or subcortical regions. Because of small number of patient (n = 5) with a cortical lesion, subgroup analysis was not carried out to examine whether lesion location results in differences in TEPs. Fifth, only M1 stimulation was examined in the current study, limiting the generalization of our findings to other brain regions. Last, given there were no associations between neurophysiologic outcomes and level of motor impairments, the behavioral significance or biomarker potential of the stroke-specific differences is limited in the current study.

## Conclusions

The present study extends our understanding of the neurophysiological state of patients with chronic stroke, especially those with quick-frequency TEP waveforms. We concluded that patients with chronic stroke with mild motor impairment showed a reduction in the cortical excitability of the ipsilesional M1 and an impairment of intra/interhemispheric connectivity. Moreover, the intracortical inhibition after stroke is complex, characterized by long duration, but low magnitude, and its clinical significance related to motor functions is awaiting more studies. The N100 of TEPs is associated with both excitatory and inhibitory functioning, as well as with certain network features, which indicate that it could be a potential biomarker in post-stroke motor recovery. MEP-based measures are limited to the investigation of the corticospinal tract, while concurrent TMS-EEG extends the utility of TMS to the investigation of underlying post-stroke intra and intercortical networks.

## Electronic supplementary material

Below is the link to the electronic supplementary material.

Additional File 1: Intracortical and intercortical networks in patients after stroke: A concurrent TMS-EEG study

## Data Availability

Data and scripts will be available by the corresponding author upon reasonable request.

## Abbreviations

AUC: area under the curve
cSP: cortical silent period
EEG: electroencephalography
EMG: electromyogram
ERSP: event-related spectral perturbation
GMFP: global mean field power
ICF: intracortical facilitation
LICI: long-interval intracortical inhibition
LMFP: local mean field power
M1: primary motor cortex
MEPs: motor-evoked potentials
PCI-st: perturbational complexity index-state transitions
RMT: resting motor threshold
SICI: short-interval intracortical inhibition
TEPs: TMS-evoked potentials
TMS: transcranial magnetic stimulation
