# Supplementary material for: Intracortical and intercortical networks in patients after stroke: a concurrent TMS-EEG study
Source: J Neuroeng Rehabil. 2023 Aug 2;20:100. doi: 10.1186/s12984-023-01223-7 (PMC10398934; doi:10.1186/s12984-023-01223-7)
Supplement: Supplementary file 1 — Additional File 1: Intracortical and intercortical networks in patients after stroke: A concurrent TMS-EEG study [file 12984_2023_1223_MOESM1_ESM.docx]

**Supplementary Materials**

**Intracortical and intercortical networks in patients after stroke: A concurrent TMS-EEG study**

Zhongfei Bai^1, 2^, Jiaqi Zhang^1^, Kenneth N. K. Fong^1^

^1^ Department of Rehabilitation Sciences, The Hong Kong Polytechnic University, Kowloon, Hong Kong SAR.

^2^ Department of Rehabilitation, Shanghai YangZhi Rehabilitation Hospital (Shanghai Sunshine Rehabilitation Centre), School of Medicine, Tongji University, Shanghai, China

**Correspondence:** Kenneth N. K. Fong, Department of Rehabilitation Sciences, The Hong Kong Polytechnic University, Kowloon, Hong Kong SAR.

Email: [rsnkfong@polyu.edu.hk](mailto:rsnkfong@polyu.edu.hk); telephone number: +852 2766 6716

| Methods | Page 2 |
| --- | --- |
| Comparisons between the bilateral hemispheres of the healthy controls | Page 4 |
| Control conditions | Page 5 |
| Fig. S1 | Page 10 |
| Fig. S2 | Page 12 |
| References | Page 13 |

# **Methods**

## *EMG data analysis*

Electromyogram (EMG) signals were offline-preprocessed by using EEGLAB 14.1.2 [1] and in-house scripts in MATLAB (The MathWorks, Inc., Natick, MA). First, data acquired by the bipolar channels for the EMG measurements were retained. Second, a Butterworth bandpass filter (fourth-order, 10 Hz – 2 kHz) was applied. Third, the continuous data were segmented into individual trials (−1000 – 999 ms relative to transcranial magnetic stimulation [TMS] trigger) and baseline-corrected (−500 – −20 ms). Fourth, cortical silent period (cSP) and motor-evoked potential (MEP) amplitudes were identified from individual trial recordings. Trials with MEP amplitudes < 50 μV were excluded. A cSP was defined as the time from TMS pulse onset to the first point of a 5-ms window at which 50% of the EMG signal data samples returned to a level at least three-fold standard deviations from the silent period. The ipsilesional cSP of eight patients was characterized by slight and persistent EMG activity after active MEPs until the EMG bursts. To determine the incomplete cSP, first, the 99% confidence interval of the root mean square of the baseline EMG was determined by means of a bootstrap resampling method (resampling repetitions: 10000). Then, the incomplete cSP was defined as a time frame that started from the TMS pulse and continued to the time when the root mean square was higher than the upper limit of the 99% confidence interval. The MEP amplitudes in the cSP measurement, termed active MEPs (aMEPs), were also quantified. The peak-to-peak amplitudes of both aMEPs and MEPs at rest were log-transformed to decrease their variability. The intracortical facilitation (ICF) and short-interval intracortical inhibition (SICI) were calculated as the ratio of MEPs produced by paired-pulse protocols to those by single pulses at rest. Finally, valid trials were averaged to obtain grand mean values for further statistical analyses.

## *EEG preprocessing*

The signals of concurrent transcranial magnetic stimulation and electroencephalography (TMS-EEG) recording were preprocessed offline by using EEGLAB 14.1.2 [1], TESA extension [2], FieldTrip [3], and custom-made MATLAB scripts, following the steps proposed by Rogasch et al. [2]. Raw signals recorded from the 59 channels were retained. Subsequently, the continuous signals were segmented into individual trials (−2000 – 1999 ms) and baseline-corrected (−500 – −10 ms). The trials with large artifacts or noise, identified by visual inspection, were excluded. Bad channels were also excluded if they were malfunctioning due to the refractory contamination of TMS-induced decay artifacts. The numbers of TMS-evoked potential (TEP) trials in patients with stroke (89.0 ± 2.0) and age-matched healthy participants (89.3 ± 1.8) were comparable. The data around TMS pulses (−2 – 15 ms) were removed and interpolated using a cubic method, followed by a down-sampling procedure to 1 kHz. Thereafter, two rounds of independent component analysis based on FastICA (systematic approach and tanh contrast function) were carried out. The first round was done to remove the largest TMS-decay artifact detected by a semi-automated component classification algorithm implemented in TESA. The EEG signals were bandpass-filtered (1 – 80 Hz) and bandstop-filtered (48 – 52 Hz) using a fourth-order Butterworth filter, followed by another segmentation from −1000 to 999 ms. The FastICA was conducted again to remove remaining artifacts (*e.g*., eye movement, persistent muscle activity, and electrode noise) by visual inspection and the semi-automated component classification algorithm. Excluded channels were interpolated back, and the reference channel was also recovered. Last, the EEG signals were referenced to the common average, and TEPs were obtained by averaging across trials. If the stroke lesion was in the left hemisphere, the EEG signals after preprocessing were left-to-right flipped for statistical comparison and visualization purposes.

# **Comparisons between the bilateral hemispheres of the healthy controls**

## *MEP-based measures*

Paired *t*-tests revealed that there were no significant differences in the RMT (*t* = −0.71, *P* = 0.486), aMEPs (*t* = −0.58, *P* = 0.571), cSP (*t* = −1.61, *P* = 0.124), MEPs (*t* = 1.08, *P* = 0.295), ICF (*t* = −0.32, *P* = 0.753), or SICI (*t* = −0.32, *P* = 0.754) between the bilateral hemispheres of the healthy controls. Therefore, the left and right measurements from the healthy controls were averaged to obtain merged values for further between-group comparisons.

## *TMS-evoked potentials*

A single-pulse stimulation to the left and right M1 produced comparable amplitudes of TEP peaks in the healthy controls, as indicated by a cluster-based permutation test. Furthermore, point-to-point paired *t*-tests did not reveal a significant difference between the GMFP of the left and right TEPs after application of the FDR correction. Likewise, comparable PCI-st (*t* = 1.38, *P* = 0.184) values were found in healthy controls.

## *TMS-related oscillatory power*

To examine whether ERSP derived from the left and right M1 stimulations differed significantly at the early and late stages in the healthy controls, we performed cluster-based permutation tests and found no significant differences in any frequency band or in any channel.

# **Control conditions**

## *Auditory-evoked potentials*

To verify whether our TMS-EEG setups (i.e., noise masking) were appropriate for eliminating the contamination of auditory-evoked potentials (AEPs) from TEPs, another two control conditions, AEPs with noise masking (AEPs MASKED) and AEPs without noise masking (AEPs NOT MASKED), were conducted in 16 out of 21 aged matched healthy controls and 21 young (right-handed; age = 28.1 ± 3.2 years; 8 females) healthy participants. The EEG system, electrode montage, sampling rate, and online filter were the same as those in the TMS-EEG recording. All procedures were conducted in a similar manner to the TMS-EEG recording, but the coil was placed 5 cm away from the scalp. Electrical field simulation indicated that this distance was largely unable to induce a valid electrical field in the brain cortex. In terms of the intensity, we chose the highest one as the reference if the RMT of the bilateral M1 was different.

## *EEG data preprocessing and analysis*

The raw signals of AEP conditions were also preprocessed using similar approaches to those used for the TEPs. After a down-sampling procedure to 1 kHz, the continuous signals were segmented into individual trials (−2000 – −1999 ms) and baseline-corrected (−500 – −10 ms). Bad trials and bad channels were also excluded after visual inspection. A TMS pulse artifact was still observable in the raw signal, but the TMS-induced decay artifact was not identified, due to lack of direct contact with the electrodes. Therefore, the TMS pulse artifact (−2 – 10 ms) was removed, and interpolation was performed using a cubic method. The same bandpass and bandstop filters for the TEPs were applied for the AEP signals, followed by another segmentation from −1000 ms to 999 ms. Then, FastICA was carried out to remove physiological and electrical artifacts. After interpolation for excluded channels, the EEG signals were referenced to the common average, and AEPs were obtained by averaging across trials. Most recently, the study by Rocchi et al. [4] suggested that TEPs within the middle (65 – 120 ms) and late (120 – 270 ms) time windows post TMS pulses were easily contaminated with AEPs if the click sound of TMS discharge was not appropriately masked, whereas the early (15 – 65 ms) TEPs were not. We therefore defined the same windows of interest in order to understand the extent to which our noise masking could eliminate AEPs from TEPs.

## *Statistical analyses*

Non-parametric cluster-based permutation tests of the Monte Carlo method were conducted to address the multiple-comparisons problem in spatial and temporal dimensions when we examined the within- and between-group differences in the AEPs [5]. We determined whether a sample (i.e., spatio-temporal pairs in the temporal domain and spatio-temporal pairs in the time-frequency domain) could be clustered by using an alpha threshold of 0.05, and at least two neighboring channels with statistical significance were required to define a cluster. Ten thousand random permutations were carried out, and *t*-values within every cluster were summed for cluster-level statistics in each permutation. The significance probability of permutation tests, also known as the *P*-value of clusters, was obtained by calculating the proportion of random permutations resulting in larger *t*-statistics than the observed one. Any positive or negative clusters with *P*-values <0.025 were considered significantly different.

To investigate the correlations among AEPs MASKED, AEPs NOT MASKED, and TEPs, Spearman rank correlation analysis was performed across channels for spatial correlation and across the timepoints within early, middle, and late time windows. For the group-level analysis on the correlation coefficient, we first transformed it to the Fisher’s *Z* value, which was submitted for further group-level statistical analysis. Then, the group-level mean of the *Z* value was transformed back to a Spearman correlation coefficient for plotting. The statistical significance of group-level spatial correlation was examined for all timepoints (*n* = 2000) using one-sample *t*-tests against zero, and FDR correction was applied. For the temporal correlation, *Z* values in the time windows were compared with the baseline for all channels (*n* = 60), using a cluster-based permutation test (number of permutations: 10,000) to address the multiple comparisons problem in the spatial dimension.

## *Results*

Fig. S1. shows the spatiotemporal characteristics of grand-averaged AEPs MASKED, AEPs NOT MASKED, and TEPs. From visual inspection, AEPs MASKED and AEPs NOT MASKED had almost completely abolished early peaks at the sensor level, whereas much large early peaks were found in the TEPs, primarily located at the TMS-stimulated site. In the middle and late time windows, AEPs MASKED were not completely suppressed by the white noise, but the amplitudes were much lower than those of AEPs NOT MASKED and the TEPs.

For the statistical comparison between AEPs MASKED and AEPs NOT MASKED, a cluster-based permutation test indicated a positive (*P* < 0.001) and a negative cluster (*P* < 0.001) over the central area in the middle and late time windows, respectively, thus suggesting that the white noise was effective in suppressing the AEPs evoked by the clicking sound while the TMS was discharging. In addition, the TEPs had significantly larger amplitudes than the AEPs MASKED in the middle time window (*P* < 0.001) and late time window (*P* < 0.001) did. Likewise, a positive cluster (*P* < 0.001) and a negative cluster (*P* < 0.001) were found in the comparison between the TEPs and the AEPs NOT MASKED. These findings suggested that neural signals evoked by the TMS and AEPs were easily mixed up in the middle and late time windows, and noise masking could suppress the contamination of the AEPs. Importantly, the TEPs had significantly larger early amplitudes than both the AEPs MASKED (*P* < 0.001) and AEPs NOT MASKED (*P* < 0.001) did, primarily located at the stimulated site. The FastICA analysis suggested that the TEPs had a unique, independent component located around the stimulated site (Fig. S1C).

Spatial correlation analysis showed that the AEPs MASKED and AEPs NOT MASKED spatially overlapped significantly, starting even from the early time window. However, the TEPs were spatially associated with the AEPs MASKED and AEPs NOT MASKED from approximately the middle time window. In terms of temporal correlations, we did not find significant clusters showing correlated amplitudes between the AEPs MASKED and TEPs in the early and middle windows. However, a large number of channels showed statistical significance in the remaining temporal correlation analyses. Taken together, these findings suggest that the TEPs had a unique early response following the TMS pulses and located specifically around the stimulated site, and that the early response was also free from the contamination of AEPs. Although the AEPs were not completely suppressed by noise masking in the middle time window, the amplitude of the TEPs was not significantly correlated with that of the AEPs MASKED, thus suggesting a modest contamination of AEPs in the TEPs. In contrast, highly correlated temporal and spatial relationships among the AEPs MASKED, AEPs NOT MASKED, and TEPs were found in the late time window, thus indicating that the clicking sound during coil discharge contributed substantially to the late response of the TEPs.


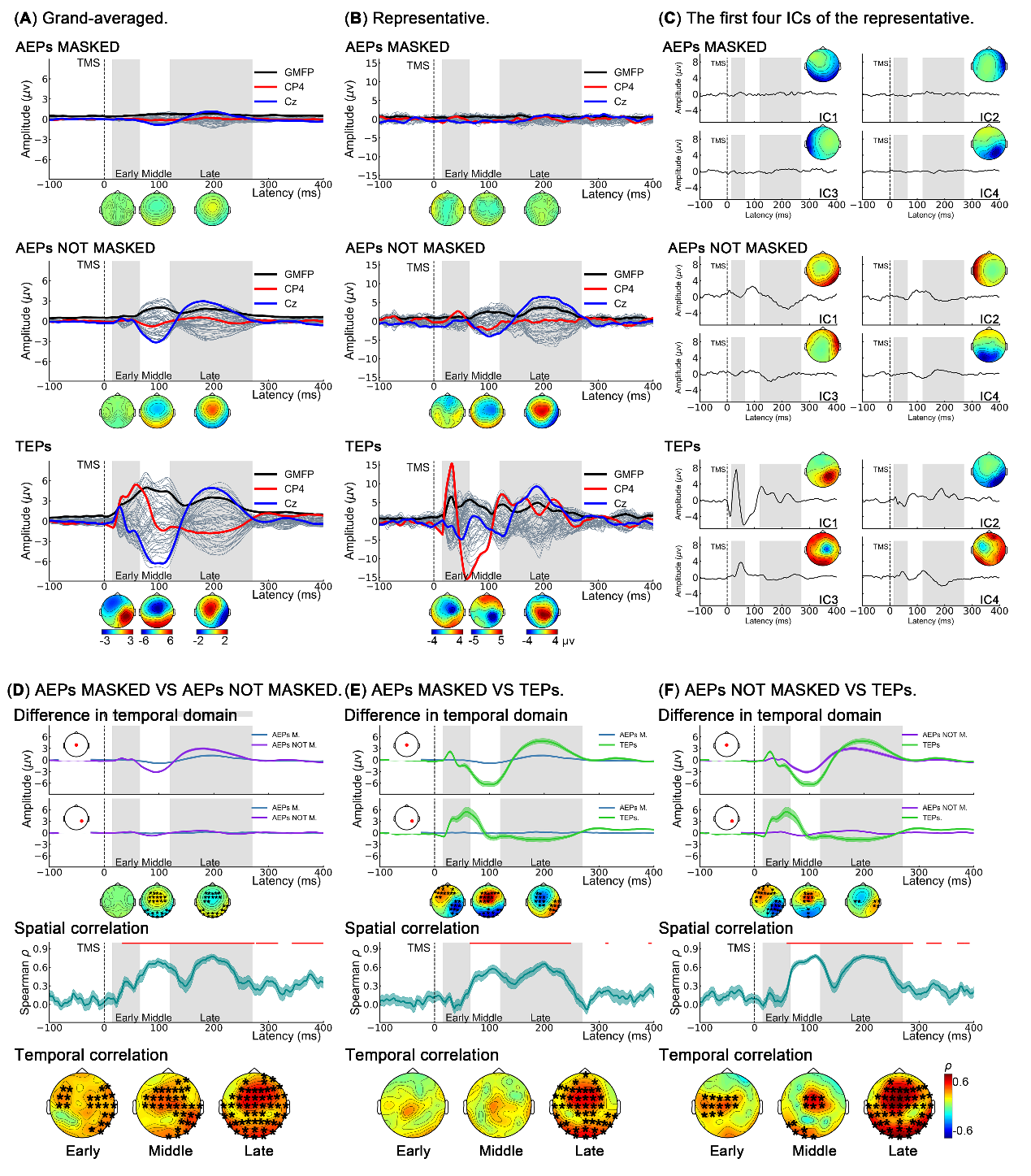


**Fig. S1** The effect of white noise in eliminating AEPs from the TEPs. (A) Butterfly plots of AEPs MASKED, AEPs NOT MASKED, and TEPs are shown. The gray-white rectangles indicate the three time windows, defined with reference to previous studies. (B) Data of a representative in each condition are shown. (C) The first four ICs of the representative healthy participant. The ICs of the representative were obtained by applying FastICA decomposition. (D – F) Respective differences in the temporal domain, spatial correlation, and temporal correlation among the three conditions. Difference in the temporal domain: Time series of two channels (Cz, CP4) are provided, located at the red dots. Spatial correlation: Averages of spatial correlation coefficients are shown. Red lines indicate time windows in which spatial correlation coefficients were still significantly different against zero after applying FDR correction. Temporal correlation: temporal correlation coefficients were calculated for early, middle, and late time windows separately, and cluster-based permutation tests were carried out to investigate whether a significant correlation existed. The asterisks (*) in the topographies represent significant clusters found in cluster-based permutation tests. The curve shadings are the means ± standard error. Note: AEPs: auditory-evoked potentials; TEPs: TMS-evoked potentials; GMFP: global mean field power; ICs: independent components.

**
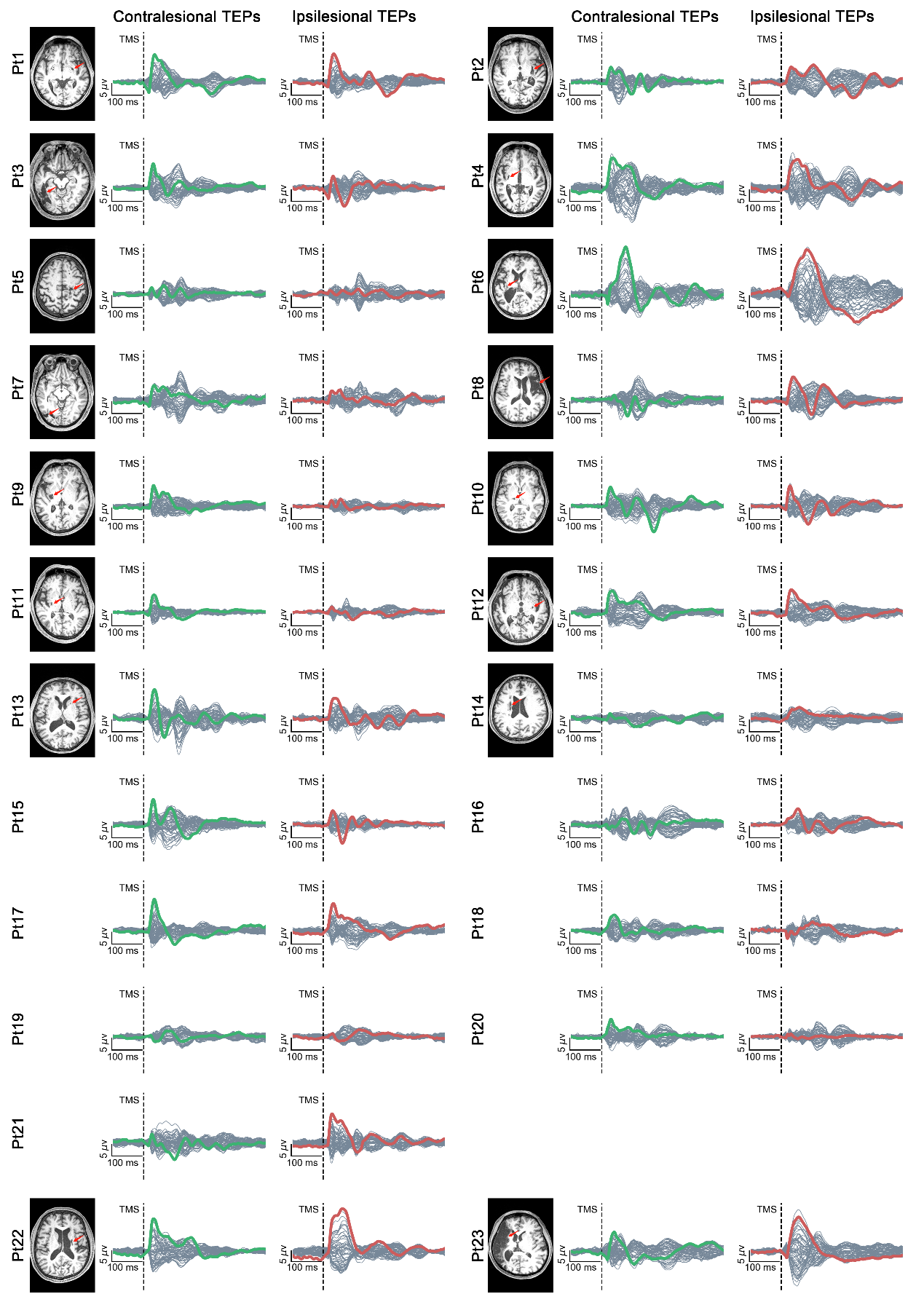
**

**Fig. S2** TEPs obtained from contralesional and ipsilesional stimulation in our patients with stroke (*n* = 23). T1-weighted MR images are depicted; red arrows indicate the location of stroke lesions. The TEPs of the last two patients were characterized by slow frequency and large local amplitudes lasting from 30 to roughly 100 ms, which were similar to those obtained during non-rapid eye movement sleep. Note: TEPs: TMS-evoked potentials.

# **References**

1. Delorme A, Makeig S. EEGLAB: an open source toolbox for analysis of single-trial EEG dynamics including independent component analysis*.* J Neurosci Methods. 2004;134(1):9-21.

2. Rogasch NC, Sullivan C, Thomson RH, Rose NS, Bailey NW, Fitzgerald PB, Farzan F, Hernandez-Pavon JC. Analysing concurrent transcranial magnetic stimulation and electroencephalographic data: a review and introduction to the open-source TESA software*.* Neuroimage. 2017;147:934-51.

3. Oostenveld R, Fries P, Maris E, Schoffelen JM. FieldTrip: Open source software for advanced analysis of MEG, EEG, and invasive electrophysiological data*.* Comput Intell Neurosci. 2011;2011:156869.

4. Rocchi L, Di Santo A, Brown K, Ibáñez J, Casula E, Rawji V, Di Lazzaro V, Koch G, Rothwell J. Disentangling EEG responses to TMS due to cortical and peripheral activations*.* Brain Stimul. 2021;14(1):4-18.

5. Maris E, Oostenveld R. Nonparametric statistical testing of EEG- and MEG-data*.* J Neurosci Methods. 2007;164(1):177-90.
